# Fish Pathology Research and Diagnosis in Aquaculture of Farmed Fish; a Proteomics Perspective

**DOI:** 10.3390/ani11010125

**Published:** 2021-01-08

**Authors:** Márcio Moreira, Denise Schrama, Ana Paula Farinha, Marco Cerqueira, Cláudia Raposo de Magalhães, Raquel Carrilho, Pedro Rodrigues

**Affiliations:** 1 CCMAR—Centre of Marine Sciences, University of Algarve, Campus de Gambelas, 8005-139 Faro, Portugal; mjmoreira@ualg.pt (M.M.); dschrama@ualg.pt (D.S.); aresende@ualg.pt (A.P.F.); macerqueira@ualg.pt (M.C.); csraposo@ualg.pt (C.R.d.M.); rvcarrilho@ualg.pt (R.C.); 2 University of Algarve, Campus de Gambelas, 8005-139 Faro, Portugal; 3 IPMA—Portuguese Institute for the Sea and Atmosphere, EPPO—Aquaculture Research Station, Av. Parque Natural da Ria Formosa s/n, 8700-194 Olhão, Portugal

**Keywords:** proteomics, fish diseases, aquaculture, fish pathology, fish welfare

## Abstract

**Simple Summary:**

The objective of this review is to provide readers with a state-of-the-art description of the main factors affecting farmed fish pathologies and its diagnoses. A special focus is given to the use proteomics technologies as a tool in the evaluation of pathogens and host-pathogen interactions and its impact in disease characterization and control.

**Abstract:**

One of the main constraints in aquaculture production is farmed fish vulnerability to diseases due to husbandry practices or external factors like pollution, climate changes, or even the alterations in the dynamic of product transactions in this industry. It is though important to better understand and characterize the intervenients in the process of a disease outbreak as these lead to huge economical losses in aquaculture industries. High-throughput technologies like proteomics can be an important characterization tool especially in pathogen identification and the virulence mechanisms related to host-pathogen interactions on disease research and diagnostics that will help to control, prevent, and treat diseases in farmed fish. Proteomics important role is also maximized by its holistic approach to understanding pathogenesis processes and fish responses to external factors like stress or temperature making it one of the most promising tools for fish pathology research.

## 1. Introduction

The demand for animal protein for human consumption is rising as a result of an exponential increase in the world population. Aquaculture is becoming an increasingly important source of protein available for human consumption since is an industry capable of providing solutions to feed a rapidly growing human population and reduce poverty in many countries [1,2,3]. To achieve that, the scale of aquaculture production and the range of farmed species has increased dramatically over the last two decades [4]. Live production always comprises a risk for loss due to infectious diseases [5], with farmed fish, due to husbandry practices in aquaculture, being more vulnerable than wild fish to diseases from a wide range of bacterial, viral, parasitic and fungal infections [6]. Also, the tendency to higher density production systems, the perturbations in ecological systems balance related to pollution and climatic changes, and the expected increase in international transactions of aquaculture products and their derivatives contributed to alterations on the dynamics of interaction between organisms, infectious agents, and people. This influences pathogen rates of replication and proliferation, leading to a broader geographic distribution of pathogenic agents and an increase in species affected by disease outbreaks [7,8]. This makes disease outbreaks an important constraint to this industry, with a significant impact on the quality, safety and volume of the fish produced throughout the world [9,10,11,12], that can lead to market access exclusion and major economic loss or costs to the producer [8,13,14].

**Figure 1:**
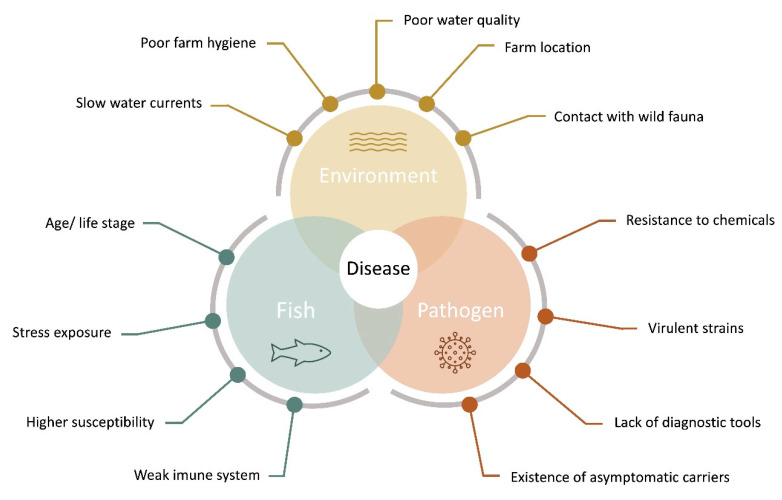
Aquaculture disease diagram, indicating the main factors for the evaluation of pathogen, and host-pathogen interactions intervening in fish disease outbreaks (adapted from [19,20]).

For several authors, disease outbreaks in aquaculture are the result of a complex network of interactions on aquatic systems between the produced organism, several environmental and zootechnical aspects, and possible pathogenic agents, that present a series of unique challenges in aquatic organism’s health [15,16,17,18,19], as represented in Figure 1.

To address infectious pathologies in farmed fish, approaches like epidemiological studies on main areas of aquatic animal health as transboundary and emerging aquatic animal diseases, animal health surveillance and biosecurity program development should be performed. These are crucial to disease prevalence monitorization, early detection of emerging exotic and new diseases and quality management improvement of aquaculture operations [15,18,19,21].

Nevertheless, to obtain proper epidemiological models, animal health surveillance and biosecurity programs must integrate environmental information and information from different areas like pathogenesis, disease diagnosis, disease resistance, physiological response to pathogens, pathogen characterization, host immune system responses characterization, disease biomarkers and organism response to disease treatment products [22,23].

The amount of data from different origins and an increase in the reported frequency and severity of marine diseases demands that new diagnostic tools should be implemented for a more rapid and effective diagnosis [24,25,26]. Thus, several scientific advances in aquatic health continue to close the gap to veterinary medicine, and new optical, analytical chemistry, molecular biology [27], and Omics techniques are becoming a reality that offers a vast array of benefits to the aquaculture industry [12,28]. Proteomics techniques are one of those new tools, and one of the most interesting approaches for health management, epidemiology, and fish disease research [3,22,23,29,30]. Proteomics refers to the methodology that addresses the study of the entire complement of proteins expressed in a specific state of an organism or a cell population [31,32]. The proteome, or the full protein complement of the genome, is a highly structured entity, where proteins exert their cellular functions with specificity in time and location, in physical or functional association with other proteins or biomolecules [33,34]. High-throughput proteomics methods based on mass spectrometry (MS) allow the measurement of multiple properties for thousands of proteins, including their abundance, tissue distribution, sub-cellular localization, post-translational modifications and protein-protein interactions [34]. Proteomics-based approaches can therefore offer unique insights into fish cellular regulation in response to pathogens and during disease progression, besides enabling fast and sensitive pathogen detection and identification.

In this manuscript, detailed information regarding the use of proteomics in several disease aspects, with a special focus on the role of stress and welfare in disease, and the importance of pathogen identification and host-pathogen interactions on disease diagnostics and characterization, will be provided.

## 2. Fish Health, Stress and Welfare

Despite being the most consumed animal, fish are seldom afforded the same level of concern regarding their welfare as other vertebrates. The scientific research around fish welfare is at an early stage compared with other land animals produced for human consumption [35]. In part, this lack of consideration is due to the gap between public perception of their intelligence and the scientific evidence [36], along with the absence of a unified definition of the concept [37]. Nevertheless, most definitions consider mainly a feelings-based and a function-based approach [38]. The first gives regard to the emotional-like state of the animal, while good welfare is defined as the absence of negative feelings and the presence of positive feelings [39]. The second definition is more focused on the biological, physiological and health perspective of the animal, while good welfare is defined as the fish’s ability to cope and adapt to its environment while maintaining homeostasis [40]. Although the fish’s health state offers objective criteria as part of a welfare assessment, it does not provide the complete picture. Good health is essential to ensure good welfare, however, it does not necessarily indicate that the fish is in a good welfare state [37]. On the other hand, poor health i.e., the reduced ability of the animal to normal functioning, to cope with stressful conditions and to prevent disease, generally implies/leads to a bad welfare status in a variety of contexts. For example, deceased fish, as a consequence of disease, constitute a source of infection and compromise water quality [41]. Additionally, chemical treatments for specific outbreaks can also trigger some level of disturbance on the fish [42,43]. Importantly, a healthy animal in an optimal welfare environment can also be suddenly struck by an acute infection reducing its welfare. For instance, in the case of fish produced in cages, pathogens are naturally embedded in the environment [44]. In most cases, it is often the lousy welfare status itself, due to poor husbandry conditions, which translates into impaired health. Thus, in summary, health and welfare are intimately linked, and poor welfare can be interpreted both as a cause and a consequence of poor health. This section focuses on health as a cornerstone for fish welfare assessment and the effects of stressors on disease resistance, reviewing the most recent approaches employed to study the relationship between certain diseases/pathologies and welfare.

**Figure 2:**
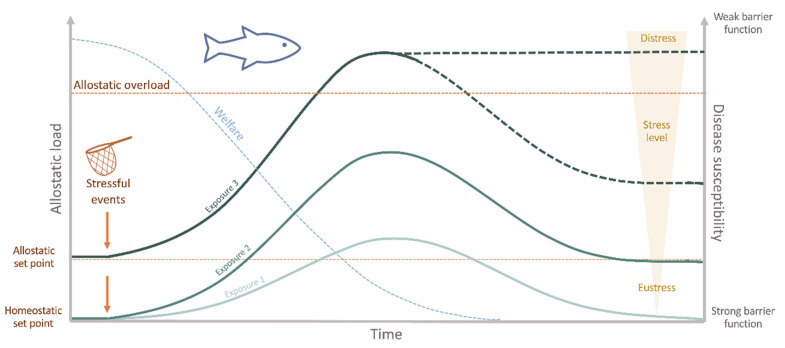
Interaction between welfare, allostatic load, disease susceptibility and the repetitive/chronic stressful experiences appraised by the fish. Stressful stimuli may induce either adaptive (eustress) or maladaptive allostasis (distress). If the stressor persists, recovery to the original homeostatic state (homeostatic set point) may be incomplete. In this case, a newly defined set point for future adaptation is settled (allostatic setpoint). As a result, the welfare status is decreased with time and stress experienced. The cumulative burden of adaptation (allostatic load) is thus constituted by the beneficial stressful events that the fish can cope with, while the allostatic overload represents the state when stress overcomes the organism’s natural regulatory capacity, which may induce a state of no-recovery. At this step, primary barrier function is severely impaired increasing disease susceptibility, which may cause illness and ultimately death.

In aquaculture, inappropriate husbandry conditions, or even standard farming practices, are everyday stressors in culture systems [45]. The allostatic load imposed on the animals can reduce functioning immune mechanisms, consequently favoring diseases and threatening fish welfare (Figure 2). For instance, drastic changes in water temperature (from 27 °C to either 19–23 °C or 31–35 °C) decreased the immune response and resistance to pathogens in Mozambique tilapia (*Oreochromis mossambicus*) [46]. More recently, using a transcriptomics approach, the rearing density in Nile tilapia (*Oreochromis niloticus*) was shown to significantly impact on the susceptibility to the oomycete *Saprolegnia parasitica* [47]. However, the association between husbandry-induced stress and disease is not that straightforward. For example, acute stressors have been reported to enhance [48,49,50,51] or decrease [52,53] some innate immune responses in fish. On the contrary, chronic stressors have mainly been indicated as immunosuppressors [54,55,56,57,58]. From a productivity perspective, the health of the fish is often interpreted as “absence of disease”, since from either an ethical or an economic point of view, any disease state is unacceptable for the industry [44]. Therefore, disease prevention and eradication are crucial aspects of a fish farm to ensure the production’s sustainability. Providing optimal welfare conditions, monitoring the health parameters routinely and alleviating stress are necessary steps towards this goal.

Stress is considered a state of threatened homeostasis [59], which is re-established by a complex network of changes in the physiological systems (allostasis) [60]. As in all other vertebrates, in the face of a perceived stressor, fish launch a widespread reaction, the so-called physiological stress response, which allows the individual to adjust and cope with the predictable and unpredictable changes in its surroundings (eustress) [61]. As a primary response, cortisol and catecholamines are released into the bloodstream, which will induce a series of downstream reactions [62]. In fact, stress is not necessarily detrimental nor immediately equates compromised welfare. Instead, in the short term, it is an essential adaptation to ensure the best chances of survival [37]. However, when reaching an allostatic overload, usually as a result of a prolonged, repeated and/or unavoidable stressor, maladaptive effects such as impaired growth and/or reproductive and immune functions, arise (Figure 2) [63,64]. In this case, these are largely associated with diminished welfare and may jeopardize fish health and survival (distress) [65]. The questions raised here are the cost of this acclimation and why stress increases diseases’ susceptibility in fish. First, in terms of energetic costs, the adaptive physiological response needed to counteract the disrupted homeostasis requires a significant amount of energy. This means that if part of the fish’s energy is allocated to face the challenge, then fewer resources will be available for other energy-demanding biological functions, such as some mechanisms of the defense repertoire: the epithelial barriers and the immune system [44]. In terms of immune responses, several mechanisms are immediately activated to respond directly to the challenge. These include an increase of inflammatory markers, the release of hormones and the expression of acute-phase proteins [66]. Even if a fish has managed to adapt to the stressor for a certain period, these energy stores will eventually be depleted if the stressor persists. Consequently, the total consumption of energy reserves gives rise to the allostatic overload, and the fish may no longer be able to adapt, which can lead to immunosuppression, disease, and in the case of more severe disturbances, even death (Figure 2) [63]. Moreover, several studies also demonstrated the impact of stressful husbandry conditions on the functioning of the epithelial barriers, i.e., the mucus and the epidermal surfaces of the skin, gills and intestine, which constitute the primary lines of defense against pathogens and harmful substances, showing that injury of these barriers, inevitably leads to impaired disease resistance [67]. Changes in these barriers have been reported in Atlantic salmon (*Salmo salar*), Atlantic cod (*Gadus morhua*) and rainbow trout (*Oncorhynchus mykiss*) subjected to different acute stressors [68,69]. Moreover, in Atlantic salmon reared under low dissolved oxygen levels, impaired intestinal barrier function was also observed [70]. These disturbances have mainly been associated with high cortisol levels, though various other hormones, such as catecholamines, endogenous opioids, pituitary hormones, and serotonin, intervene here [71]. Indeed, it is known that cortisol plays an immunomodulatory role, inhibiting specific constituents of the immune system and enhancing others, such as induction of apoptosis, change of differentiation patterns, inhibition of cytokine release and inhibition of immunocyte migration [72,73,74,75]. Nevertheless, the cortisol response may vary among different species and even among individuals (coping styles) [76] and be affected by several other parameters (e.g., domestication level, age, nutritional state, stressor severity, among others) [53,77,78,79], which may obscure the relationship between stress and immune status. A detailed description of how the endocrine-immune response is mounted and the mechanisms behind these immunoregulatory changes is out of the scope of this review, for this, the authors refer to recent publications [66,80].

Deepening our scientific knowledge on the mechanisms relating to stress, fish health and welfare, is paramount for the sustainable aquaculture industry. In recent years, more advanced high-throughput technologies, as the case of proteomics, started to be successfully employed in aquaculture research, including for the study of fish diseases and welfare, providing a holistic understanding of the molecular events underlying the physiological stress response and valuable insights on the differential proteins involved in inflammatory processes and immune responses [30,58,81]. Proteomic studies on fish target mainly the liver, however, blood plasma and mucus are taking crescent importance, mainly from an immunological point of view, as skin mucus is one of the primary barriers of defense in fish [82,83,84,85,86] and plasma acts as a mirror/reporter of physiological or pathological conditions [87,88]. Important applications of proteomics in this field concern the study of the effects of certain diseases and parasites on the proteins’ abundance and modifications and the investigation of the host-pathogen interactions [88,89,90,91,92,93,94,95]. For example, joint studies evaluating changes in the proteome of fish challenged with a specific pathogen after exposure to a rearing stressor are scarce. However, the existing proteomic studies demonstrating aquaculture and environmental stressors clearly modulating the fish’s immune function [58,96,97] reveal that these technologies are already promising sensitive approaches to study this relationship.

## 3. Disease Diagnostics

**Figure 3:**
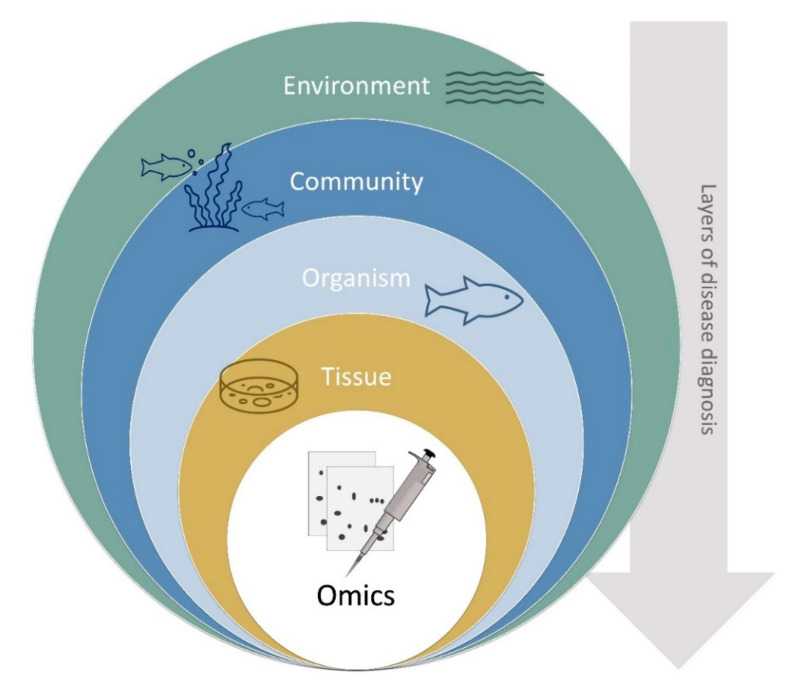
Disease diagnosis concentric ring, representing layers of disease diagnoses as environment, community, organism, tissue, and omics as a tool to interpret cell/tissue responses (adapted from [26]).

To properly diagnose pathology in aquaculture, we must consider disease as a problem with multiple levels of increasing biological complexity, ranging from environmental to the cell, genome and proteome level (Figure 3) [26,27].

New areas like Proteomics can be an important complement to more classical approaches like pathogen identification, disease symptomatology and histopathological analysis to achieve a good disease diagnosis in aquaculture [22,23,27,29,30]. In Proteomics, regardless the complexity of the analysed protein mixtures that can range from hundreds, to several thousands of proteins, the major goal is the accurate identification of the highest number of proteins as possible in those mixtures [32]. In gel-based approaches, proteins are first separated by one (1-DE)—or two-dimensional gel electrophoresis (2-DE) and then identified by mass spectrometry, whereas in gel-free approaches (or MS-based) protein mixtures remain in solution prior to protein identification. In each case, protein samples may be digested to peptides by a sequence-specific enzyme, typically trypsin, in a so-called peptide-based “bottom-up” proteomics approach, to distinguish it from the analysis of entire proteins in “top-down” proteomics. Peptide samples can then be separated and analysed by liquid chromatography coupled to tandem mass spectrometry (LC-MS/MS), usually employing electrospray ionization (ESI) as the method to convert the peptides to gas phase ions for MS analysis. Alternatively, peptide samples can be analysed by matrix-assisted laser desorption/ionization (MALDI) time-of-flight (TOF) mass spectrometry. The method of choice will always depend on the main research objective, costs and expertise, with MALDI-TOF MS based strategies being most suited for microbial identification and diagnosis, as a rapid, sensitive and economical in terms of both labour and costs [98]. On the other hand, LC-MS/MS is most suited for large-scale, systematic characterization of proteomes, e.g., involved in host-pathogen interactions, allowing multiplex sample analysis and quantitation. In the following sections we will discuss in more detail main applications of proteomics in pathogen characterization and in host-pathogen interactions.

### 3.1. Pathogen Identification

Pathogen identification is a key area in disease diagnosis and management. Classical, immunological and molecular methods have been routinely and extensively used to address this area [26]. However, in the last ten years proteomics has emerged as a powerful tool for pathogen identification, strain typing and epidemiological studies [98], as can be observed in Table 1.

This powerful tool can be used for pathogen identification as a complement to other molecular genetic techniques, being Matrix-assisted laser desorption/ionization time-of-flight mass spectrometry (MALDI-TOF-MS) the main technique used for this purpose [98]. Is also very useful for virulence factors characterization and life cycle characterization of pathogens [125,126].

### 3.2. Symptomatology

Pathogens have different impacts on fish since the severity of infection depends on diverse factors, such as the host species, fish age and physiological state, environmental conditions, and disease stage [127,128].

Generally, diseases can be expressed in different stages and can develop from an acute to a chronic disease or the reverse way. This is the case of the infectious salmon anaemia (ISA) in Atlantic salmon outbreaks, with initial low mortality, causing minor alterations in the fish (e.g., anaemia). This chronic stage can go unnoticed if diagnostic measures are not performed. Acute disease stages with high mortality may occur sporadically, increasing the severity of the disease (e.g., ascitis and haemorrhages). Furthermore, ISA chronic infection develops in the autumn, while the acute stage is observed more in the spring [129].

Besides infections with virus, bacteria, parasites and fungus, fish can be exposed to secondary infections that can aggravate their health status and increase mortality rate [130]. Observation of clinical signs (external or internally) and behaviour alterations can help to detect a pathogen presence in fish. However, the signs exhibited in response to a disease can be non-specific of that disease and very similar between different pathogen infections (Table 2). Moreover, the fish might show few or none of these signs. After these observations, gross and microscopic pathology can be used to confirm some pathogens yet is often necessary the use of more specific types of diagnosis for the identification [19].

As shown in Table 2, even if disease symptomatology is extremely used in disease characterization, it is difficult to distinguish between several diseases with similar symptomatology. Taking this into account, several researchers suggested that host-pathogen interaction are more reproducible and more reliable indicators for disease diagnostics [21,81,168].

## 4. Tools for the Study of Host-Pathogen Interactions

### 4.1. The “Holobiome” Approach: Metagenomics and Metaproteomics

The host-pathogen interactions are extremely complex and can be established at multiple levels, ranging from molecular, cellular and physiological, to populations and ecosystems levels [169]. The host-pathogen interaction starts when the host organism is challenged by a pathogenic agent e.g., virus, bacteria, prion, fungus, viroid, or parasite, thus triggering a biological response; the pathogen, in turn, develops a back-fighting response [170,171]. This interaction implies induction of gene expression and protein synthesis on both sides, and an infectious process may develop in the host, leading ultimately to death, if the host response or defense system fails to combat the pathogenic challenge [171]. However, a wider perspective on host-pathogen interactions may be undertaken [172], spanning these interactions to the associated microbial populations e.g., the host microbiome, known as the “holobiome” approach [173]. Indeed, it has been demonstrated that the microbiota may play a critical role in the immune response of organisms [174]. The “holobiome” approach on the study of fish host-pathogen interactions i.e., between the fish host, its microbiome, the pathogen, and other environmental microorganisms, has been pointed as a critical aspect for further development of rational strategies aiming at fish disease prevention and resistance [172]. Moreover, this holistic knowledge of fish host-pathogen interactions could contribute to promote sustainability in aquaculture, by reducing the use of antibiotics, responsible for a negative environmental impact of this industry [172].

Metagenomics and metaproteomics are among the most powerful and emerging high-throughput tools in marine/ocean environments to disclose the genome and proteome, of the associated microbial communities [172,175,176,177]. These methodologies are still scarce on aquaculture research, although it might be extremely useful in the study of microbial populations inherent to the farmed fish surrounding environment. Furthermore, metagenomics and metaproteomics approaches enable the characterization of the microbiota associated to fish skin mucous or fish gut, thus unravelling key genes or proteins in the immune function, that may act as the whole biosystem through complex networks during fish host-pathogen interactions. An additional and major benefit of these tools is the possibility of accessing to unculturable species, the vast majority of disease-related microbes in aquaculture, whose identity and function would otherwise remain unknown [172].

### 4.2. Omics-Based Strategies and Protein-Protein Interaction (PPI) Networks

The knowledge on the genes/proteins and metabolites involved in host-pathogen interactions during infectious events has assisted to considerable advances in the last years, due to the implementation of high-throughput technologies like RNA-sequencing (RNA-Seq) [178] mass-spectrometry based proteomics [126] and metabolomics [179]. On the other hand, the combination of omics-based approaches with in vivo studies, addressing interactions from the single cell to the whole animal level, by using zebrafish (*Danio rerio*) larvae as infection models [180,181], constituted a step forward in the understanding of the cellular mechanisms that occur during fish-pathogen interactions.

The large-scale proteome characterization from both pathogen(s) and fish host, either in health or disease conditions, allowed to identify proteins with a major role in disease defense mechanisms (recently reviewed by [126]), whose regulatory complexity might be represented by protein-protein interaction (PPI) networks. The integration of proteomics with other omics-based approaches may be used to model networks capable of predicting the interaction dynamics between cellular bio-components involved in fish-pathogen immune responses (e.g., DNA, RNA, protein, metabolite) to foster new therapeutic strategies in aquaculture [27,179]. It can be stated that proteins as the main key players and building blocks across all life forms, since they catalyze and control virtually all cellular processes [33], hence occupy a central role in host-pathogen interactions. PPIs networks, either determined at experimental level e.g., through interactome proteomic approaches [182] or predicted by computational methodologies, are gaining increasing popularity and becoming one of the most useful tools in the understanding of pathogenesis [183]. PPI networks may offer unique insights into host-pathogen and pathogen co-infection interactions, by identifying effective health/disease biomarkers, thus accelerating the implementation of prevention measures, treatment of fish diseases and vaccination development [183]. PPI network analysis will be no doubt, one of the most powerful and cost-effective tools to assist in fish disease management in the aquaculture sector.

In sum, there is a significant number of emerging tools to address fish host-pathogen interactions that can help in the control, prevention, and treatment of diseases in farmed fish, becoming evident that these interactions are extremely complex, requiring integrated, complementary, and holistic approaches to be fully understood.

Proteomics is also highly used to understand the fish immune response, surviving strategies of the pathogen and interactions between fish and pathogen [126]. As this technique can show differential expression of identified proteins in various stages of fish development, and different conditions of feeding, stress and disease [184], it provides a holistic overview of several functions of the fish metabolism [185]. Differential expression of proteins affected by any pathogen might be studied using gel-based (1 or 2-DE) or gel-free applications (LC-MS/MS) [186]. An overview of some proteomic studies with fish pathogens is shown in Table 3. In the case of viruses, several proteins have been modified although differences depend on the type of virus. Spleen tissue of infected zebrafish and turbot (*Scophtalmus maximus*) with *Megalocytivirus* showed that cytoskeletal and cellular signal transduction proteins were modified in both species [89,187]. Pancreas disease caused by salmonid alphavirus in Atlantic salmon showed that humoral components of the serum were affected during the first weeks after infection [94]. Proteins involved in the glycolytic pathway and cytoskeleton were modified during viral haemorrhagic septicaemia rhabdovirus in zebrafish [188]. Host defences against spring viremia of carp virus use mainly proteins like vitellogenin and grass carp reovirus induced protein Gig2 [189]. These proteins seem to have a potential antiviral activity. Red blood cells in teleost can respond to pathogens and trigger an immune response against the viral septicaemia haemorrhagic virus [190]. As a defensive mechanism against cyprinid herpesvirus-2 several proteins like herpes simplex infection pathway, *p53* signalling pathways and phagosome pathway were induced [191]. Against bacterial infections, the immune system of teleost fish is triggered, as shown by both the induced acute phase and immune responses in the liver or spleen, respectively of rainbow trout against *Aeromonas salmonicida* [192,193]. Or by the enhanced immune response against *Aeromonas hydrophila* in common carp (*Cyprinus carpio*) and zebrafish [91,194]. More specific, proteins involved in the cellular stress response were modified in channel catfish (*Ictalurus punctatus*) after a challenge with *Edwardsiella ictaluri* [195]. Enteric redmouth disease in salmonids resulted in several differentially expressed proteins in head kidney and liver samples of rainbow trout like antioxidants, lysozyme, metalloproteinase, cytoskeleton and c-type lectin receptor proteins [95]. Up-regulated proteins involved in peptidase and hydrolase activity, lysosome and metabolic pathways were identified in intestinal mucosal samples [196]. Detected on the first defence barrier of fish, the skin mucus showed differentially expressed proteins of the immune system of Atlantic cod with vibriosis [83]. Also, by proteins like heat-shock proteins, cathepsins and complement components it is shown that the immune response is up-regulated against *Streptococcus parauberis* in olive flounder (*Paralichthys olivaceus*) [197]. Mitochondrial enzymes also showed altered expression upon *Moraxella* sp. infection in kidney tissues of gilthead seabream (*Sparus aurata*) [198]. Infections with the ciliated parasite *Ichthyophthirius multifiliis* results in increased mucus secretion in fish. Proteomics of mucus in infested common carp with *I. multifiliis* showed an up-regulation of immune-related and signal transduction proteins in the first defence barrier of fish [199]. Infestations of Atlantic salmon with the ectoparasite *Lepeophtheirus salmonis* were studied on fish mucus and detected an increase in proteins involved in proteolysis [82]. When looking into the plasma of infested gilthead seabream with *Amyloodinium ocellatum*, differences were found in proteins involved in the acute-phase response, inflammation, homeostasis and wound healing but, in this case, the innate immunological system was not activated [88]. Another ectoparasite that affects Atlantic salmon is the amoeba *Neoparamoeba perurans*, causing amoebic gill disease. Proteomic analysis showed that proteins involved in the cell cycle regulation, inflammation pathway, oxidative metabolism and immunity were affected [200,201].

Although some examples were given in Table 3, more studies were performed as each tissue/organ in fish represents a specific barrier against pathogens, and several of them have been used in proteomic studies. Like the shotgun proteomic approach of serum proteins from turbot infected by *Edwardsiella tarda*, showing that immunoglobulins and complement component proteins were important antimicrobial proteins [202]. Or the study on Infections by *Mycrocystis aeruginosa* infections on medaka (*Oryzias latipes*) fish, that showed differences in liver proteins such as stress response, lipid metabolism and developmental processes [203].

Proteomics may also be used to analyse the pathogen in vitro, which is shown by the reduced expression of proteins related to the tricarboxylic acid cycle and chemotaxis when chlortetracycline antibiotic was used against *A. hydrophila* [204]. Virulence mechanisms of bacteria can be studied using proteomics for the visualization of up and down-regulated proteins in virulent and avirulent strains. In the case of *E. tarda* proteins, like antigenic protein Et 46, bifunctional polymyxin resistance protein and iron-cofactored superoxide dismutase type I were identified [92]. And in the case of *Y. ruckeri* proteins like anti-sigma regulatory factor, arginine deiminase, and superoxide dismutase Cu-Zu were identified [107]. It is known that different conditions like temperature may affect a facultative pathogen like *Pseudomonas plecoglossicida* which showed upregulation of the pyoverdine protein at 18 °C, which is important for bacterial multiplication [205]. The iron metal is essential for bacteria [27], as shown in *Vibrio* spp., which was able to trap iron [125], and by *Aeromonas salmonicida* [206]. The outer membrane proteins, important for virulence by *Y. ruckeri* on Atlantic salmon and rainbow trout were identified in different isolates [109]. As parasites go through various life stages different proteins are needed in each one of them. Proteomics was applied to identify these proteins in *I. multifiliis* and showed proteins involved in biological processes, cellular components, molecular functions, binding and catalytic activity [207]. And in the case of *Anisakis simplex* proteins like pseudocoelomic globin, endochitinase 1 and paramyosin were identified in L3 developmental stage [208].

To understand the interaction between a pathogen and its host proteomics seems to be a good tool. As mentioned before the outer membrane proteins are important for pathogenicity. The immunity of fish might be reduced as proteins of bacteria are capable of interacting with extracellular proteins [209]. In the case of Gram-negative bacteria, outer membrane proteins seem to be able to survive inside the fish [210] and can present resistance to antimicrobial peptides [211]. Another technique used to identify pathogenic proteins was immunoproteomics. Immunized sera from rohu (*Labeo rohita*) and grass carp (*Ctenopharyngodon idella*) was used to identify outer membrane proteins from *E. tarda* [212] and *Flavobacterium columnare* [213]. Several outer membrane proteins were identified by immunized sera of Nile Tilapia with *Francisella noatunensis* subsp. *orientalis* [211].

## 5. Conclusions

Overall, we can look at proteomics as a very promising tool for fish pathology research and diagnostic, allowing a more holistic approach to pathogenesis processes, giving important information on pathogen identification and virulence mechanisms characterization and in host-pathogen interactions, enlightening new stress response routes and previously unknown physiological host responses.

However, the use of proteomics in fish aquaculture is still in its early days and limited to some sequenced organisms. Further progress in defining aquacultural proteomes and large-scale datasets from diseased fish and fish pathogens will boost the use of proteomic techniques in aquaculture, that will lead to new and exciting discoveries on this field.

But one of the most promising and interesting areas and one that we believe being the future trend in further understanding the fish response to pathogens, is the study of the interaction holobiome-host-pathogen, with a strong potential for new and more detailed and integrated knowledge of fish pathogenesis.

## Figures and Tables

**Table 1:** Resume of some of the proteomic techniques applied to pathogen identification, characterization, and virulence.

Pathogen	Disease	Pathogen ID	Metabolic Process	Proteomic Method	Reference
**Vírus**					
Singapore grouper iridovirus (SGIV)	Sistemic diseases	Vírus proteín characterization	-	SDS-PAGE MALDI-TOF-MS	[99]
Singapore grouper iridovirus (SGIV)	Sistemic diseases	Vírus envelope proteín analysis and characterization	-	1-DE MALDI-TOF/TOF-MS/MS and LC-MALDI-TOF/TOF-MS/MS	[100]
Cyprinid herpesvirus 3 (CyHV-3)	Koi herpesvirus (KHV) disease	Functional analyses of the virion transmembrane proteome	-	SDS-PAGE LC-MS/MS	[101]
**Bacteria**					
***Photobacterium damselae*** **subsp. *piscicida***	Pasteurellosis	-	Importance of outer membrane proteins in osmotic adaptations and bacterial virulence	2-DE LC-nano ESI-Q-TOF MS/MS	[102]
***Photobacterium damselae*** **subsp. *damselae***	Vibriosis	-	Importance of iron in virulence	2-DE MALDI-TOF/TOF-MS	[103]
***Photobacterium damselae***	Pasteurellosis	Differentiation of *Photobacterium damselae* subspecies	-	MALDI-TOF-MS	[104]
***Edwardsiella tarda***	Haemorrhagic septicaemia	Identification of proteins associated with virulent and avirulent strains	-	2-DE MALDI-TOF MS	[105,106]
***Edwardsiella tarda***	Haemorrhagic septicaemia	-	Virulence determinants	2-DE ESI MS/MS	[92]
***Yersinia ruckeri***	Enteric redmouth disease	Identification and characterization of biotype 1 and biotype 2 strains	-	Nano LC-MS/MS	[107]
***Yersinia ruckeri***	Enteric redmouth disease	-	Importance of iron in virulence	Nano LC-MS/MS	[108]
***Yersinia ruckeri***	Enteric redmouth disease	-	Importance of outer membrane proteins in bacterial virulence	Gell free and 1-D nLC-ESI-MS/MS	[109]
***Flavobacterium columnare***	Columnaris disease	-	Virulence determinants	2-D LC ESI MS/MS and 2-DE MALDI TOF/TOF MS	[110]
***Flavobacterium psychrophilum***	Bacterial coldwater disease	Proteomic profiling of strains, the importance of iron in virulence	-	2-DE LC-MS/MS	[111]
***Flavobacterium psychrophilum***	Bacterial coldwater disease	Differentiation of *Flavobacterium psychrophilum* from *Flavobacterium psychrophilum*-like species	-	MALDI-TOF MS	[112]
***Vibrio parahaemolyticus***	Vibriosis	-	Identify proteins regulating antimicrobial peptide resistance	2-DE LC-ESI-Q-TOF MS/MS	[113]
***Streptococcus agalactiae***	Streptococcosis	-	Temperature effects on bacterial virulence	NanoUPLC-HDMS^E^	[114]
***Streptococcus iniae*** **and *Streptococcus parauberis***	Streptococcosis	Identification and molecular fingerprinting	-	MALDI-TOF-MS	[115]
***Streptococcus parauberis***	Streptococcosis	Identification of bacterial strains	-	MALDI-TOF-MS	[116]
***Vagococcus salmoninarum***	Cold-water streptococcosis	Typing and characterization of bacterial strains	-	MALDI-TOF-MS	[117]
***Streptococcus iniae***	Streptococcosis	Proteomic profile of a pathogenic strain	-	2-DE MALDI-TOF-MS	[118]
***Piscirickettsia salmonis***	Salmonid rickettsial syndrome	Detection and identification	Virulence determinants	MALDI-TOF-MS	[119]
***Tenacibaculum sp.***	Tenacibaculosis	Identification of *Tenacibaculum* species	-	MALDI-TOF-MS	[120,121]
***Mycobacterium marinum***	Mycobacteriosis	Identification of *Mycobacterium marinum* subspecies	-	MALDI-TOF-MS	[122]
**Several bacterial species**	-	Differentiate several gram-negative fish pathogenic bacteria	-	MALDI-TOF-MS	[123]
**Fungus**					
***Saprolegnia parasitica***	Saprolegniasis	Identification and characterization of developmental stages	-	iTRAQ SDS-PAGE Nano-LC-MS/MS	[124]
**Parasites**					
***Caligus rogercresseyi***	Sea lice	Detection and identification	Virulence determinants	MALDI-TOF-MS	[119]

**Proteomic techniques abbreviations—1-DE:** One-dimensional Electrophoresis; **2-DE:** Two-dimensional Electrophoresis; **SDS-PAGE:** Sodium Dodecyl Sulphate-Polyacrylamide Gel Electrophoresis; **iTRAQ:** Isobaric Tag for Relative and Absolute Quantitation; **MALDI-TOF-MS:** Matrix-Assisted Laser Desorption and Ionization Time-of-Flight Mass Spectrometry; **MALDI-TOF/TOF-MS:** Matrix-Assisted Laser Desorption and Ionization (Time-of-Flight)^2^ Mass Spectrometry; **MALDI-TOF/TOF-MS/MS:** Matrix-Assisted Laser Desorption and Ionization (Time-of-Flight)^2^ tandem Mass Spectrometry; **LC-MALDI-TOF/TOF-MS/MS:** Automated Liquid Chromatography Matrix-Assisted Laser Desorption and Ionization (Time-of-Flight)^2^ tandem Mass Spectrometry; LC-MS/MS: Liquid Chromatography tandem Mass Spectrometry; **ESI MS/MS:** Electrospray Ionization tandem Mass Spectrometry; **LC- ESI-Q-TOF MS/MS:** Liquid Chromatography Electrospray Ionization Quadrupole Time-of-Flight tandem Mass Spectrometry; **LC-nano ESI-Q-TOF MS/MS:** Liquid Chromatography and Nano-Electrospray Ionization Quadrupole Time-of-Flight tandem Mass Spectrometry; **LC-ESI-MS/MS:** Liquid Chromatography Electrospray Ionization tandem Mass Spectrometry; **nLC-ESI-MS/MS:** Nano-scale Liquid Chromatography Electrospray Ionization tandem Mass Spectrometry; **NanoUPLC-HDMS^E^:** Ultra-Performance Liquid Chromatography with High Definition tandem Mass Spectrometry.

**Table 2:** Symptomatology of important diseases caused by virus, bacteria, and parasites.

Disease	Pathogen	Host	Clinical Signs/Pathology	References
**Virus**				
Epizootic haematopoietic necrosis (EHN)	Epizootic haematopoietic necrosis virus	Redfin perch (*Perca fluviatilis*), Rainbow trout (*Oncorhynchus mykiss*)	Erratic swimming, darkened skin, skin ulcers, exophthalmia, swollen spleen and kidney, petechial haemorrhages on fins, ascites, abdominal distension	[131]
Infectious haematopoietic necrosis (IHN)	Infectious haematopoietic necrosis virus	Salmonids	Lethargy, darkened skin, exophthalmia, eye haemorrhage, pale gills, swollen abdomen, opaque faecal casts, petechial haemorrhage on fins, visceral pallor	[132,133]
Infectious Pancreatic Necrosis (IPN)	Infectious pancreatic necrosis virus	Salmonids	Irregular swimming, loss of appetite, darkening of the skin, distended abdomen, exophthalmia, pale gills petechial haemorrhages, visceral ascites, intestine with catarrhal exudate and pale liver	[134,135,136]
Infectious salmon Anaemia (ISA)	Infectious Salmon Anaemia virus	Atlantic salmon (*Salmo salar*)	Lethargy, anaemia, exophthalmia, pale gills and internal organs, ascites, oedemas, petechiae in visceral fat, liver and spleen congestion	[137,138]
Lymphocystis disease	*Lymphocystis* disease virus	Broadly infectious	Nodular lesions on the skin, fins and internally	[137]
Pancreatic disease (PD)	Salmonid alphaviruses (SAV)	Rainbow trout (*Oncorhynchus mykiss*), Atlantic salmon (*Salmo salar*)	Lethargy, hang in the corners of the cage or rest in the bottom, loss of appetite, yellow faecal casts, ascites, petechial haemorrhages in pyloric caecal fat, lesions in pancreas and skeletal and cardiac muscle	[139,140]
Viral haemorrhagic septicaemia (VHS)	Viral haemorrhagic septicaemia virus	Broadly infectious	Aberrant swimming (spiral, leaping, flashing), exophthalmia, darkened skin, anaemia, pale gills and liver, internal haemorrhages, ascites leading to a swollen abdomen, swollen and hyperaemic kidney	[137,141]
Viral nervous necrosis (VNN)	Betanodaviruses	Broadly infectious	Lethargy, abnormal swimming; anorexia, skin darkening, abdominal distension, hyperinflation of the swim bladder	[142,143]
**Bacteria**				
Vibriosis	*Vibrio anguillarum*	Broadly infectious	Lethargy, cease feeding, darkened skin, exophthalmia and corneal opacity, pale gills, petechiae at fin bases and skin, ulcers, generalized septicaemia	[127,144,145]
Pasteurellosis	*Photobacterium damselae* subsp. *piscicida*	White perch (*Morone americanus*) yellowtail (*Seriola quinqueradiata*) gilthead seabream (*Sparus aurata*)	Darkened skin, swollen spleen, white-spotted lesions in spleen and kidney, bacterial accumulations on the tissues of internal organs	[146,147]
Furunculosis	*Aeromonas salmonicida* subsp. *salmonicida*	Broadly infectious	Lethargy, cease feeding, darkened skin, exophthalmia, haemorrhages at the base of the fins, enlarged spleen, lesions on the skin (furuncles), ulcers, pale liver, general septicaemia	[148,149]
Tenacibaculosis	*Tenacibaculum maritimum*	Broadly infectious	Flashing swimming behaviour, anorexic, erosion on the skin, fins (tail rot), head and gills, petechial haemorrhage on the abdominal peritoneum, ulcers	[150]
Bacterial gill disease (BGD)	*Flavobacterium branchiophilum*	Coldwater fish (mainly salmonids)	Loss of appetite, gill infestation, increased opercular movements, gasping at the water surface, respiratory distress	[151]
Rainbow Trout Fry Syndrom (RFTS)	*Flavobacterium psychrophilum*	Salmonids	Lethargy, anorexia, distended abdomen, darkened skin in caudal peduncle area, skin ulceration, swollen spleen, pale organs	[152]
Red spot disease (Winter disease)	*Pseudomonas anguilliseptica*	Eels, salmonids, gilthead seabream (*Sparus aurata*), European seabass (*Dicentrarchus labrax*), European cod (*Gadus morhua*)	Erratic swimming, petechial haemorrhages in the skin and liver, distended abdomen, ascitic fluid in the peritoneal cavity, pale liver, haemorrhaged kidney, intestine with yellowish exudate	[144,153]
**Disease**	**Pathogen**	**Host**	**Clinical Signs/Pathology**	**References**
Lactococcosis	*Lactococcus garvieae*	Broadly infectious	Lethargy, anorexia, exophthalmia, distended abdomen, ascitic fluid in the peritoneal cavity, congestion and haemorrhages of liver, intestine, spleen and kidney, haemorrhagic septicaemia	[154]
**Parasites**				
Amoebic gill disease (AGD)	*Neoparamoeba perurans*	Atlantic salmon (*Salmo salar*), rainbow trout (*Oncorhynchus mykiss*)	Lethargy, respiratory distress, increased opercular movements, whitish patches on gills and excessive mucus	[155,156]
White spot disease/Ichthyophthiriasis	*Ichthyophthirius multifiliis*	Freshwater fish	Hyperactive (initially), lethargic; pale gills, darkened skin, white spots in the skin, increase of mucus production, skin ulcers, frayed fins, pale liver, enlarged spleen and kidney	[128,157,158]
White spot disease/Cryptocaryoniasis	*Cryptocaryon irritans*	Saltwater fish	Hyperactive (initially), lethargic, numerous small whitish spots on the skin surface, petechial haemorrhages on the skin, excessive mucus production, skin ulcers, corneal clouding and blindness	[128,159]
Amyloodiniosis	*Amyloodinium ocellatum*	Broadly infectious	Cease feeding, scratch against the bottom, infested gills and skin, excessive mucus production, epithelial erosion on attachment sites	[160,161,162]
Trichodinosis	*Trichodina* sp.	Broadly infectious	Lethargy, cease feeding, infested gills, skin and fins, Greyish colour due to excessive mucus production, skin lesions in attachment sites, frayed fins	[163,164]
Gyrodactilosis	*Gyrodactylus salaris*	Salmonids	Infest mainly fins and skin, lethargy, anorexia, emaciated fins, darkened skin, epithelium lesions	[165,166]
Sea lice	Lepeophtheirus salmonis	Salmonids	Skin lesions, especially on the head, haemorrhages, scale loss, oedema, hyperplasia and cellular inflammations	[167]

**Table 3:** Summary of some modified proteins identified by proteomics in fish infectious diseases.

Aetiological Agent	Species	Tissue	Modified Protein Groups	Reference
**Vírus**				
Infectious spleen and kidney necrosis virus (ISKNV) (*Megalocytivirus*)	Zebrafish (*Danio rerio*)	Spleen	Cytoskeletal proteins, stress response, lipoprotein and carbohydrate metabolism, signal transduction, proteolysis, metabolic and catabolic processes	[89]
*Megalocytivirus*	Turbot (*Scophtalmus maximus*)	Spleen	Cytoskeleton proteins, molecular biosynthesis, cellular signal transduction and chaperone proteins	[187]
Salmonid alphavirus subtype 3 (SAV3)	Atlantic salmon (*Salmo salar*)	Serum	Humoral components of immunity	[94]
Rhabdovirus	Zebrafish (*Danio rerio*)	Fins	Proteins of the glycolytic pathway and cytoskeleton components	[188]
Spring viremia of carp virus	Zebrafish (*Danio rerio*)	Plasma	Vitellogenin and Gig2	[189]
Viral septicemia hemorrhagic virus	Rainbow trout (*Oncorhynchus mykiss*)	Red blood cells from blood and head kidney	Proteins related to viral transcription	[190]
Cyprinid herpesvirus-2	Crucian carp (*Carassius carassius*)	Head kidney	Cytoskeleton, transport, immunologic, intracellular and physiologic proteins	[191]
**Bacteria**				
*Aeromonas salmonicida*	Rainbow trout (*Oncorhynchus mykiss*)	Liver	Complement system and acute phase response proteins	[192]
*Aeromonas salmonicida*	Rainbow trout (*Oncorhynchus mykiss*)	Spleen	Immune system, signaling molecules and interaction	[193]
*Aeromonas hydrophila*	Common carp (*Cyprinus carpio*)	Intestinal mucosa	Proteins involved in stress and immune response	[194]
*Aeromonas hydrophila*	Zebrafish (*Danio rerio*)	Gills	Stress and immune response	[91]
*Edwardsiella ictaluri*	Channel catfish (*Ictalurus punctatus*)	Head kidney	Macrophage function, cellular stress response, cellular energy production and metabolism	[195]
*Yersinia ruckeri*	Rainbow trout (*Oncorhynchus mykiss*)	Head kidney and spleen	Immune system, cellular, metabolic, developmental, multicellular, adhesion, regulation and response to stimulus	[95]
*Yersinia ruckeri*	Rainbow trout (*Oncorhynchus mykiss*)	Intestine	Metabolic process biological regulation, cellular processes and component organization	[196]
*Vibrio anguillarum*	Atlantic cod (*Gadus morhua*)	Mucus	Proteins involved in the immune system	[83]
*Streptococcus parauberis*	Olive flounder (*Paralichthys olivaceus*)	Kidney	Proteins involved in immune response, cellular recovery and glycoprotein synthesis	[197]
*Moraxella sp.*	Gilthead seabream (*Sparus aurata*)	Kidney	Mitochondrial proteins, cellular response to oxidative stress, infection and inflammation	[198]
**Parasites**				
*Ichthyophthirius multifiliis*	Common carp (*Cyprinus carpio*)	Mucus	Proteins involved in the immune and inflammatory response	[199]
*Lepeophtheirus salmonis*	Atlantic salmon (*Salmo salar*)	Mucus	Proteins involved in glycolysis, peptide synthesis, immune and defence response	[82]
*Amyloodinium ocellatum*	Gilthead seabream (*Sparus aurata*)	Plasma	Proteins involved in the acute-phase response, inflammation, lipid transport, homeostasis and wound healing	[88]
*Neoparamoeba perurans*	Atlantic salmon (*Salmo salar*)	Gill and skin mucus	Proteins involved in cell to cell signalling and inflammation pathway	[189]
*Neoparamoeba perurans*	Atlantic salmon (*Salmo salar*)	Gill and skin mucus	Proteins involved in cell cycle regulation, cytoskeletal regulation, oxidative metabolism and immunity	[201]

## Data Availability

Not applicable.
